# Recent advances in adjuvant systemic treatment for breast cancer: all systems go!

**DOI:** 10.3747/co.v13i5.130

**Published:** 2006-10

**Authors:** P.N. Fishman, S. Verma

**Affiliations:** * Department of Medical Oncology, Toronto Regional Cancer Centre, University of Toronto, Toronto, Ontario

**Keywords:** Breast cancer, adjuvant, treatment

## 1. INTRODUCTION

Breast cancer is the most common life-threatening malignancy in Western women, and the second-most common cause of cancer-related death. Estimates suggest that, in 2006, 22,185 Canadian women will be diagnosed with breast cancer and 5277 will die of the disease 1. Most breast cancers are diagnosed when surgical resection is still an option, and yet many patients still develop recurrent disease. In an attempt to prevent recurrence, adjuvant systemic therapy and radiation therapy may be offered following surgical resection.

Adjuvant systemic therapy refers to the administration, after primary surgery, of hormone therapy, chemotherapy, or trastuzumab (Herceptin: Genentech, San Francisco, CA, U.S.A.), a monoclonal antibody directed against her2/*neu*. Adjuvant treatment is intended to eliminate or delay the appearance of occult micro-metastatic disease, which is believed to be responsible for distant treatment failures after local therapy. The use of adjuvant treatment in combination with an effective screening strategy is believed to have contributed to a significant reduction in mortality from breast cancer in Western nations since the mid-1990s 2.

Over the last few years, significant advances have been made in adjuvant therapy for breast cancer. The present topic review focuses on those advances and on recent trends in adjuvant therapy based on pivotal clinical trials—of hormonal therapy, of chemotherapy, and of therapy with biological agents—that have had a significant impact on treatment of early breast cancer. The practical impact of these therapies on the care of breast cancer patients is also discussed. A description of key upcoming trials in early breast cancer is also presented.

## 2. DISCUSSION

### 2.1 Adjuvant Hormonal Therapy

The main objective of adjuvant hormonal therapy is to prevent breast cancer cells from receiving stimulation from endogenous estrogen. Hormonal therapy is beneficial only for patients with hormone-receptor-positive (hr+) disease—either estrogen-receptor-positive (er+) or progesterone-receptor-positive (pr+).

In the 1950s, ovarian ablation became a standard adjuvant treatment for hr+ early breast cancer 3. Gradually, ovarian ablation in postmenopausal women was replaced with pharmacologic hormonal agents, including selective estrogen receptor modulators (serms), such as tamoxifen, and aromatase inhibitors (ais).

#### 2.1.1 Tamoxifen as Adjuvant Therapy

Tamoxifen is a serm that inhibits the growth of breast cancer cells by antagonizing the effect of estrogen on the estrogen receptor. The role of tamoxifen in adjuvant treatment has been well studied and was reported in the Early Breast Cancer Trialists Collaborative Group (ebctcg) 15-year update ^4^.

In women with er+ breast cancer, 5 years of tamoxifen treatment reduced the annual risk of relapse by 41% and the annual death rate by 34% as compared with placebo. This finding equates to a 12% reduction in risk for disease recurrence (33% vs. 45%) and a 9% reduction in breast cancer–related death (26% vs. 35%) at the 15-year point. The perceived benefit of tamoxifen is independent of age, nodal status, and chemotherapy use. According to the ebctcg, 5 years of adjuvant tamoxifen use can also reduce by 40% the annual risk of developing a contralateral breast cancer.

Unfortunately, despite improvement in disease recurrence and survival rates with tamoxifen use, two thirds of women with hr+ breast cancer do not appear to respond to tamoxifen treatment 5 because of either primary or acquired resistance to tamoxifen. A number of factors may contribute to this resistance. One possibility is interaction between estrogen–er pathways and nongenomic growth-promoting pathways (“crosstalk”). In preclinical models, tumours demonstrating high levels of human epidermal growth factor receptor (her2) may be resistant to tamoxifen because of presumed enhanced crosstalk between the er and her2 pathways. Resistance may also be explained by tamoxifen’s partial agonist effects on the estrogen receptor or by chronic estrogen deprivation. In addition, relative resistance to tamoxifen may be related to inheritance of the *CYP2D6* genotype, which is associated with a reduction in the activation of tamoxifen to its active metabolite endoxifen.

Resistance to tamoxifen may explain why no additional benefit accrues to extending tamoxifen beyond 5 years 6–9. There continues to be significant controversy over the use of tamoxifen for longer durations. Two ongoing trials, the Adjuvant Tamoxifen Longer Against Shorter (atlas) and the Adjuvant Tamoxifen Treatment Offer More (attom) trials will randomize women to 5 years or more of tamoxifen. These trials may help to clarify the duration of tamoxifen use for women with hr+ early breast cancer (Table I).

The possible life-threatening complications of tamoxifen also need to be kept in mind. Tamoxifen use has been shown to increase the risk of endometrial cancer (0.5% incidence), and venous thromboemboli (3.5% incidence, including a 1.7% incidence of deep vein thrombosis or pulmonary embolism) in the prevention and treatment settings alike 5. Although these side effects are uncommon, they need to be carefully evaluated and discussed with patients in the decision-making process leading to adjuvant endocrine therapy.

#### 2.1.2 AIs as Adjuvant Therapy in Postmenopausal Women

In postmenopausal women, ais suppress plasma estrogen levels by inhibiting or inactivating the enzyme aromatase, which is responsible for synthesizing estrogen from androgens 10. Third-generation ais include the steroidal ai exemestane and the nonsteroidal ais anastrozole and letrozole.

In premenopausal women, use of ais alone is not recommended, because the reduction in estrogen feedback to the hypothalamus–pituitary axis increases gonadotropin secretion, which then stimulates the ovaries 11.

More than 30,000 postmenopausal women have been evaluated in several large randomized trials that compared ais with tamoxifen as an up-front therapy in sequence after 2–3 years of tamoxifen therapy, or with extended adjuvant therapy after 5 years of tamoxifen (Tables II and III).

##### Up-Front Therapy

Two large randomized trials compared tamoxifen with ais as initial adjuvant hormonal therapy for postmenopausal women with early breast cancer. In the Anastrozole or Tamoxifen Alone or in Combination (atac) trial, 9366 postmenopausal women with er+ or unknown receptor status breast cancer were randomized to 5 years of adjuvant tamoxifen or anastrozole, or a combination of the two 12,13. At a median follow-up of 68 months, significant improvement in disease-free survival (dfs) was noted with anastrozole as compared with tamoxifen. The study demonstrated a lower risk of recurrence (hazard ratio: 0.87; *p* = 0.01) and a longer delay for recurrence (hazard ratio: 0.79; *p* = 0.005) in patients receiving anastrozole than in those receiving tamoxifen. The 3-year dfs was 89% for anastrozole, 87% for tamoxifen, and 87% for the combination group. In a retrospective subgroup analysis, the benefit for anastrozole was more apparent in women with er+pr− receptor status (hazard ratio for breast cancer events: 0.43 with er+pr− and 0.85 with er+pr+). A 42% reduction in contralateral breast cancers was also observed in the anastrozole group. No difference in overall survival (os) was seen between the groups.

With respect to toxicity, patients in the anastrozole arm had fewer cerebrovascular events, hot flashes, vaginal bleeding, endometrial cancers, and venous thromboembolic events. However the rates of osteoporosis, bone fractures, and myalgias or arthralgias was higher with anastrozole than with tamoxifen.

The second up-front ai trial was the Breast International Group (big) 1-98 study 14. This four-arm trial randomized 8010 postmenopausal women to either tamoxifen or letrozole for 5 years or to tamoxifen or letrozole for 2 years followed by 3 years of the alternative agent. At present, only the results for the up-front arms are available. At a median follow-up of 29 months, improved event-free survival (efs) was seen in women randomized to initial letrozole (hazard ratio: 0.81; *p* = 0.003). The 5-year dfs was 84% for letrozole, and 81.4% for tamoxifen. Distant recurrences were also fewer with letrozole (hazard ratio: 0.73; *p* = 0.001). The big 1-98 study analysis did not find any differences in benefit based on receptor status. The results of the sequential treatment arms have yet to be reported; those reports are expected in 2008 (Table I)

As had been reported in previous ai studies, letrozole use was associated with a higher incidence of osteoporosis, and a lower incidence of endometrial and thromboembolic events. An increased rate of hypercholesterolemia (43% vs. 19%) was also observed as compared with the rate seen in the tamoxifen arm; however, most of the occurrences were grade i. It also appears that no absolute increase from baseline cholesterol occurred in patients on the letrozole arm, but a significant reduction in the cholesterol levels of patients receiving tamoxifen explains the difference seen in the two arms. The effect of ais on blood lipids and the possibility of an increase in cardiac events remains an important area of further research.

##### AIs Used in Sequence

Five trials have evaluated the use of ais in sequence after tamoxifen. Patients were studied after either 2–3 years of tamoxifen or 5 years of tamoxifen (extended adjuvant setting)

###### Extended Adjuvant Aromatase Inhibitors After 5 Years of Tamoxifen

The National Cancer Institute of Canada Clinical Trials Group (ncic-ctg) MA.17 study examined extended adjuvant treatment with letrozole after 5 years of tamoxifen 15–17. A total of 5187 post-menopausal women were randomized to letrozole or placebo after 5 years of tamoxifen. At a median follow-up of 30 months, dfs was superior with letrozole (hazard ratio: 0.58; *p* = 0.00008), and 4-year dfs with letrozole was 94% as compared with 90% on the placebo arm. Letrozole resulted in a 40% lower risk of distant recurrences (hazard ratio: 0.60; *p* = 0.002). Overall survival was similar between the groups (hazard ratio: 0.82; *p* = 0.3). An extension of MA.17 named MA.17R is ongoing. In the latter trial, women who have had 5 years of letrozole are randomized to 5 more years of letrozole or to placebo (Table I).

###### Sequential Aromatase Inhibitors after 2–3 Years of Tamoxifen

Four additional trials have evaluated the use of ais after 2–3 years of tamoxifen therapy, compared with completing tamoxifen for a total of 5 years of endocrine treatment.

The Intergroup Exemestane Study (ies) randomized 4742 postmenopausal women with er+ or unknown receptor status disease after 2–3 years of tamoxifen to either exemestane for 2–3 years, or a continuation of tamoxifen for a total of 5 years 18. In 122 patients, er status was originally reported as unknown and was later found to be estrogen receptor negative (er−) 19. At a median follow-up of 58 months, the hazard ratio for breast cancer recurrence in the exemestane group was 0.76 as compared with the tamoxifen group (*p* = 0.0001). Exemestane was also superior with regard to distant disease recurrence (hazard ratio: 0.83; *p* = 0.03) and reducing the risk of contralateral breast cancer (hazard ratio: 0.56; *p* = 0.04). When all the patients were analyzed for os, no differences were seen between the groups (hazard ratio: 0.85, *p* = 0.08). However, in er+ or unknown-status patients, switching to exemestane after only 2–3 years significantly improved overall survival (hazard ratio: 0.83; *p* = 0.05) 19.

In a combined analysis of the abcsg-8 trial and the German Adjuvant Breast Cancer Group (arno)-95 trial, 3224 postmenopausal women with er+ breast cancer who completed 2 years of tamoxifen were switched either to anastrozole for 3 years or continued on tamoxifen for a total of 5 years 20. At the 28-month follow-up, an improved efs was seen in women switching from tamoxifen to anastrozole (hazard ratio: 0.60; *p* = 0.0009), and 3-year efs was 96% for the anastrozole group and 93% for the tamoxifen group. In a recent update at the 2006 meeting of the American Society of Clinical Oncology (asco), arno 95 showed a hazard ratio of 0.66 (*p* = 0.049) for dfs at a median follow-up of 30 months 21. A survival advantage was also seen (hazard ratio for os: 0.53; *p* = 0.045). Importantly, however, none of the patients on this trial received adjuvant chemotherapy.

In the Italian Tamoxifen Anastrozole (ita) trial, 448 postmenopausal women with node-positive and er+ breast cancer were randomized to 5 years of tamoxifen or to anastrozole after 2–3 years of tamoxifen, for a total treatment duration of 5 years 22. At a median follow-up of 36 months, dfs (hazard ratio: 0.35; *p* = 0.001) and local recurrence-free survival (rfs hazard ratio: 0.15; *p* = 0.03) were both significantly improved in the anastrozole group.

These trials demonstrate that tamoxifen followed by an ai may reduce local and distant recurrences and improve dfs. The ies and arno 95 trials were also able to show an improvement in os with sequence treatment.

Based on the preceding studies, ais now have an integral role in the management of hr+ postmenopausal early breast cancer. An up-front strategy is generally preferred for patients deemed to be at high risk of recurrence and for those who have contra-indications to tamoxifen. Overexpression of her2/*neu* may also predict responsiveness to ai treatment, although this subject remains controversial. Also, for patients who are at high risk of recurrence and who are already on tamoxifen, consideration should be given to switching to an ai after 2–3 years of therapy.

Several clinical questions remain to be answered concerning adjuvant endocrine treatment. Many of the ongoing clinical trials will address these questions (Table I). The big 1-98 trial will address the issue of upfront ai use or switching from tamoxifen to an ai after 2–3 years of therapy. The phase iii trial MA.27, which is comparing anastrozole with exemestane, and the phase iii face trial, which is comparing up-front letrozole with anastrozole, will help to determine whether the ais differ in efficacy. The soft/text and perche trials are addressing use of ais in premenopausal females with ovarian ablation. Another study, the nsabp-42, will randomize patients who have completed 5 years of upfront ai therapy or 2–3 years of tamoxifen followed by an ai, to either letrozole or placebo for 5 years. The results of those trials will be valuable in guiding future treatment practices. An important question that remains to be answered is how nonsteroidal and steroidal ais might be used in sequence for adjuvant therapy.

### 2.2 Adjuvant Chemotherapy

A number of trials reported over the last few years have established the role of adjuvant chemotherapy in early breast cancer (Table IV). The 2000 ebctcg overview found an increased survival benefit with polychemotherapy as compared with no adjuvant chemotherapy ^4^. In women younger than 50 years of age, combination chemotherapy reduced the annual risk of relapse by 40%, and the annual risk of death by 30%—a 10% improvement in 15-year absolute survival (42% vs. 32%). In women 50–69 years of age, combined chemotherapy reduced the annual risk of recurrence by 20%, and the annual risk of death by 12%. Those reductions represent a 3% improvement in 15-year absolute survival (50% vs. 47%).

#### 2.2.1 Anthracycline-Containing vs. CMF-Containing Chemotherapy

The ebctcg overview compared anthracycline-based regimens with cmf (cyclophosphamide, methotrexate, and 5-fluorouracil)–based regimens and found an 11% reduction in the annual risk of recurrence and a 16% reduction in the annual death rate with anthracycline-based regimens ^4^. The 10-year absolute os with anthracycline-containing chemotherapy was 4% better than with cmf-containing regimens. In the nsabp-B23 trial, 2008 women with node-negative and er− breast cancer were randomized to 4 cycles of ac (doxorubicin, cyclophosphamide) as compared with 6 cycles of cmf^23^. The dfs at 5 years was equivalent between these regimens at 83%, and the os was 90.2% with ac and 88.5% with cmf (*p* = 0.76).

Certain subgroups of women may be more responsive to anthracycline-based chemotherapy. In the randomized controlled MA.5 trial, 710 premenopausal women with node-positive breast cancer received either cef (cyclophosphamide, epirubicin, 5-fluorouracil) or cmf chemotherapy 24. In that trial, patients with her2-amplified breast cancer achieved a superior benefit with cef chemotherapy (rfs hazard ratio: 0.52; *p* = 0.003; os hazard ratio: 0.65; *p* = 0.06) 25. In patients without her2 amplification, cef did not improve rfs or os (rfs hazard ratio: 0.91; *p* = 0.49; os hazard ratio: 1.06; *p* = 0.68). Amplification of her2 in breast cancer cells is therefore associated with clinical responsiveness to anthracycline-containing chemotherapy. Guidelines from asco support the use of anthracycline regimens, particularly in women who overexpress her2/*neu*. However, low levels of expression should not exclude patients from anthracycline-containing regimens 26.

#### 2.2.2 Taxane-Based Regimens

In addition to anthracyclines, taxanes have been studied quite extensively in the adjuvant setting. The two taxanes that have been studied in this setting include paclitaxel and docetaxel.

##### Paclitaxel

Three randomized trials have looked at the addition of paclitaxel to anthracycline-based chemotherapy. The Cancer and Leukemia Group 9344 trial (calgb 9344) included 3121 pre and postmenopausal women with node-positive breast cancer 27. The trial examined whether increasing the dose of anthracyclines improved survival, and if the addition of paclitaxel was beneficial. Women in the study were first randomized to ac chemotherapy (at varying doses of doxorubicin) for 4 cycles; they were later randomized to receive either 4 cycles of paclitaxel or no further treatment. Women who were hr+ received 5 years of tamoxifen after chemotherapy. The 5-year dfs improved with the addition of paclitaxel to ac chemotherapy (to 70% from 65%, *p* = 0.001), and the 5-year os was also improved (to 80% from 77%, *p* = 0.009). Escalating doses of doxorubicin did not improve dfs or os. The nsabp B-28 trial randomized 3060 women with node-positive breast cancer to ac for 4 cycles with or without sequential paclitaxel for 4 cycles 28. Women who were hr+ received concurrent tamoxifen with chemotherapy. An increase in 5-year dfs was observed with the addition of paclitaxel to ac chemotherapy (76% vs. 72%, *p* = 0.006), but the 5-year os was similar between the groups (85%, *p* = 0.46). The differences between the results of nsabp B-28 and calgb 9344 may be explained by the concurrent administration of tamoxifen and chemotherapy in the nsabp trial, which may lead to reduced effectiveness of the chemotherapy.

The MD Anderson trial randomized 524 patients to 8 cycles of fac (5-fluorouracil, doxorubicin, cyclophosphamide), or 4 cycles of paclitaxel and then 4 cycles of fac. At the 4-year time point, no significant differences were found between the two groups in terms of dfs and os. The imbalance in chemotherapy between the two arms may have contributed to the lack of findings in this trial.

Based on these foregoing studies, there appears to be a small survival advantage of adding paclitaxel to anthracycline-based chemotherapy.

##### Docetaxel

Two large randomized trials have evaluated the benefits of adding docetaxel to anthracycline-based chemotherapy. The Breast Cancer International Research Group (bcirg) study 001 randomized 1491 women with node-positive breast cancer to 6 cycles of fac or 6 cycles of tac (docetaxel, doxorubicin, cyclophosphamide) 29,30. After a median follow-up of 55 months, the 5-year dfs in the tac group was 75%, as compared with 68% in the fac group (*p* = 0.001). The 5-year os was 87% with tac and 81% with fac (*p* = 0.008). Neutropenia and febrile neutropenia rates were significantly higher with tac. Hematopoietic growth factors were not routinely administered with tac, but they were required if an episode of febrile neutropenia occurred. A second large randomized trial, pacs 01, assigned 1999 women with node-positive breast cancer to either 6 cycles of fec 100 (fluorouracil, epirubicin, cyclophosphamide) or 3 cycles of fec 100 every 3 weeks followed by 3 cycles of docetaxel every 3 weeks 31. The 5-year dfs (78% vs. 73%, *p* = 0.01) and os (91% vs. 87%, *p* = 0.01) were significantly improved with the addition of docetaxel. There were also fewer cardiac events in the patients who received docetaxel, and fewer leukemia events were reported. Febrile neutropenia was slightly higher in patients who switched to docetaxel (4.6% vs. 1%, *p* = 0.001). In a subgroup analysis, the benefit of adding docetaxel to anthracycline-based chemotherapy was greater in women over 50 years of age (older than 50 hazard ratio: 0.67; *p* = 0.001; younger than 50 hazard ratio: 0.98; *p* = 0.690).

#### 2.2.3 Dose-Dense Chemotherapy

Dose-dense chemotherapy refers to chemotherapy treatment cycles that are administered at shorter intervals than usual and hence require the use of hematopoietic growth factors. The calgb 9741 trial used a 2×2 factorial design, and compared sequential docetaxel (× 4 cycles), followed by paclitaxel (× 4 cycles), followed by cyclophosphamide (× 4 cycles) with concurrent ac (× 4 cycles), followed by paclitaxel (× 4 cycles), administered every 2 or 3 weeks 32. Patients on the dose-dense arm (chemotherapy every 2 weeks) received Filgrastim on days 3–10. A total of 2005 women with node-positive breast cancer were randomized to one of the four arms in this study. The dose-dense arms had a significantly better 4-year dfs (82% vs. 75%, *p* = 0.01) and 4-year os (92% vs. 90%, *p* = 0.013). No difference in dfs or os was observed between the concurrent and sequential dose-dense arms. The 5-year follow-up results showed that er− patients benefited from dose-dense therapy more than did er+ patients, with a statistically significant improvement seen in dfs (*p* = 0.01) and os (*p* = 0.04). The survival results in the er+ subset were not statistically significant 33. Ongoing trials such as MA.21 and nsabp B-38 are comparing other dose-dense regimens with conventional chemotherapy regimens (Table I).

The above trials demonstrate a dfs and os benefit of adding taxanes to anthracycline-based regimens. Taxane-containing adjuvant chemotherapy should be the standard of care in women with lymph node–positive breast cancer. Limited data are available on taxane-containing regimens in node-negative breast cancer; however, in high-risk node-negative women, adjuvant taxanes may be considered.

### 2.3 Adjuvant Trastuzumab

Approximately 20%–25% of breast cancers have amplification or overexpression of the gene encoding a cell-surface molecule called her2/*neu*. Overexpression or amplification of this cell surface receptor is predictive of benefit from trastuzumab (Herceptin), a monoclonal humanized antibody directed against this receptor. Trastuzumab has been shown to be beneficial in combination with chemotherapy, as compared with chemotherapy alone, for metastatic breast cancer 34. The benefit seen in the metastatic setting led to the study of this agent in the adjuvant setting. Table V summarizes the adjuvant trastuzumab trials.

#### 2.3.1 North American Studies

The nsabp B31 trial randomized 1736 women with her2/*neu*-positive and node positive breast cancer to one of two arms. In one arm, ac chemotherapy for 4 cycles was followed by 4 cycles of paclitaxel alone or paclitaxel for 4 cycles in combination with trastuzumab, followed by weekly trastuzumab for 1 year of total therapy 35.

The North Central Cancer Treatment Group (ncctg)–coordinated Intergroup trial N-9831, which studied trastuzumab in sequence to ac and paclitaxel, also evaluated sequential versus concurrent use of trastuzumab 36. In that trial, 1615 women with her2/*neu*-positive and node-positive or high-risk node-negative breast cancer received ac × 4 and were then randomized to one of the following arms:

- Weekly paclitaxel for 12 weeks
- Weekly paclitaxel for 12 weeks, and then sequential trastuzumab for 52 weeks
- Weekly paclitaxel as given in the first two arms, plus concurrent trastuzumab, and then 40 weeks of trastuzumab alone

The results of the combined analysis of these two trials, with a median follow-up of 2 years, reported a 4-year dfs with sequential trastuzumab of 86%, as compared with 67% without trastuzumab (hazard ratio: 0.50; *p* = 0.0005). The 4-year os with sequential trastuzumab was 91%, as compared with 87% without trastuzumab (hazard ratio: 0.67; *p* = 0.015). Ongoing analysis will attempt to investigate the impact of sequential or concurrent trastuzumab with paclitaxel.

Trastuzumab cardiotoxicity (chronic heart failure or cardiac death) was a concern, but the risk was increased only by 3.3% in the nsabp B31 trial (4.1% in the concurrent trastuzumab arm vs. 0.8% in the control arm). Similarly, in the ncctg N-9831 trial, the cardiac event rate with sequential trastuzumab use was 2.2%; it was 3.3% in the concurrent trastuzumab arm and 0% in the control arms.

#### 2.3.2 HERA Trial

The hera trial randomized 5090 women with her2/*neu*-positive breast cancer for observation or trastuzumab for 1 or 2 years after completion of adjuvant chemotherapy. Interim analysis for 3387 patients (1693 controls, 1694 who received trastuzumab for 1 year) revealed a 3-year dfs of 80.6% for the 1-year trastuzumab group and 74% for the control group (hazard ratio: 0.63; *p* ≤ 0.0001) 37. In the trastuzumab group, 3-year os was 92.4%; it was 89.2% in the control arm. Asymptomatic heart failure (ejection fraction less than 50%) occurred in 7% of patients in the trastuzumab group and in 2.2% in the control group. Severe heart failure occurred in 0.5% in the trastuzumab group and 0% in the control group.

#### 2.3.3 bcirg 006 Trial

In the bcirg 006 trial, 3222 women with her2/*neu*-positive, node-positive or high-risk node-negative breast cancer were randomized to ac followed by docetaxel with or without trastuzumab, or to a non-anthracycline arm (docetaxel/carboplatin and trastuzumab) 38. After 23 months of follow-up, dfs was better in the trastuzumab arms (ac/docetaxel/trastuzumab hazard ratio: 0.49; *p* < 0.0001; docetaxel/carboplatin/trastuzumab hazard ratio: 0.61; *p* = 0.0002). No significant difference was observed between the two trastuzumab arms in this trial, but trastuzumab combined with a non-anthracycline may be less cardiotoxic.

The genes encoding her2 and topoisomerase iiα (*TOPO2A*) are located side by side on chromosome 17. Co-amplification of the topoisomerase iiα gene occurs in 35% of her2-positive patients and may confer responsiveness to anthracycline-based therapy and a therapeutic advantage to anthracycline-based trastuzumab combinations. The her2-positive patients that are not co-amplified for topoisomerase IIα do not appear to have this same benefit and may be candidates for non-anthracycline-based regimens, thus avoiding the potential cardiotoxicity. Ongoing analysis of the data will help to determine if non-anthracycline–containing regimens combined with trastuzumab should be used as an alternative to anthracycline/trastuzumab combinations in this population.

#### 2.3.4 FinHer Trial

The FinHer trial looked at a course of trastuzumab that was shorter than the one used in previous trastuzumab trials described above 39. A total of 1010 women with node-positive or high-risk node-negative breast cancer were randomized to 3 cycles of docetaxel every 3 weeks, or vinorelbine every week for 8 cycles and then 3 cycles of fec60. The 232 women who were her2/*neu*-positive were randomized with or without trastuzumab given weekly for 9 weeks. At 3 years, rfs was better with docetaxel than with vinorelbine (91% vs. 86%; hazard ratio: 0.58 for recurrence/death; *p* = 0.005). In the subgroup that received trastuzumab, 3-year rfs was 89% vs. 78% in the non-trastuzumab group (hazard ratio: 0.42 for recurrence or death; *p* = 0.01). A trend towards improved os was noted in the trastuzumab group (96% vs. 93%; hazard ratio: 0.41; *p* = 0.07). Trastuzumab given over this short period was not associated with decreased left ventricular function or heart failure.

The above trials showed that at least 1 year of trastuzumab added to anthracycline- or taxane-containing adjuvant chemotherapy in her2/*neu*-positive women is beneficial in reducing recurrence and increasing os. Longer follow-up from these trials will better characterize the long-term toxicities, especially cardiotoxicity. As well, the question of which regimen is superior—concurrent or sequential chemotherapy with trastuzumab—awaits further trial analysis (Table I). Use of dose-dense chemotherapy with trastuzumab has not been evaluated in a phase iii study to date. The optimal trastuzumab duration also remains to be studied.

## 3. CONCLUSION

The use of adjuvant systemic therapy in early breast cancer is believed to have significantly contributed to the higher survival rates among women with this disease. This review article has summarized some of the key systemic adjuvant treatment trials and provides evidence for these conclusions:

- Five years of an aromatase inhibitor is the preferred initial therapy for postmenopausal hr+ breast cancer patients with high-risk breast cancer. Postmenopausal women who have already commenced on tamoxifen may cross over to an ai after 2 or 3 years, for a total of 5 years of therapy. In addition, sequential administration of letrozole for 5 additional years should be discussed with women who have completed 5 years of tamoxifen.
- When chemotherapy is being considered, an anthracycline-containing regimen is recommended especially in women with her2/*neu* overexpression.
- The addition of a taxane to anthracycline-containing chemotherapy should be considered in node-positive and high-risk node-negative patients.
- Trastuzumab-containing adjuvant therapy should be used in women with node-positive, her2/*neu*-overexpressing breast cancers, and in women with node-negative breast cancer with a tumour larger than 1 cm and her2/*neu* overexpression.

Many unanswered questions remain about systemic therapy for breast cancer, and future or ongoing trials may provide insight into these issues to improve patient care. We are also heading towards a new direction in breast cancer therapy with the use of genomic analysis to better stratify breast cancer risk and to help guide our therapeutic choices. A number of ongoing trials are evaluating these genomic tools and their clinical utility 40 (Table I).

New therapeutic approaches will continue to improve the outlook for women with early-stage breast cancer. Participation in clinical trials offers the best chance to advance knowledge in the realm of adjuvant systemic treatments for breast cancer; hence, ongoing accrual in adjuvant trials is necessary and should be encouraged.

## Figures and Tables

**TABLE I:** Key ongoing adjuvant systemic treatment trials

*Study*	*Description*
*Endocrine trials*	
big 1-98	Ongoing analysis will compare up-front use of aromatase inhibitors to sequential letrozole followed by tamoxifen, or vice versa Will also compare whether tamoxifen or an aromatase inhibitor should be used first Results on sequence arms expected in 2008
atlas (Adjuvant Tamoxifen Longer Against Shorter)	Randomize patients to 5 years of tamoxifen or longer
attom (Adjuvant Tamoxifen Treatment Offer More)	Randomize patients to 5 years of tamoxifen or longer
MA.17R	Extension of MA.17 Patients assigned to a further 5 years of letrozole or placebo
MA-27	Phase 3 trial comparing exemestane with anastrozole up front
face (Femara vs. Anastrozole Clinical Evaluation)	Phase 3 trial comparing anastrozole with letrozole up front in node-positive postmenopausal women
nsabp B-42	Phase 3 trial comparing 5 years of letrozole with placebo in patients who have completed 5 years of up-front aromatase inhibitor therapy or 2–3 years of tamoxifen followed by an aromatase inhibitor
*Premenopausal trials*	
soft	Patients who remain premenopausal within 6 months after chemotherapy or those for whom tamoxifen alone is considered adequate Tamoxifen vs. ofs + tamoxifen vs. ofs + exemestane
text	Patients receiving ofs from start of adjuvant treatment Triptorelin ± chemotherapy + tamoxifen vs. Triptorelin ± chemotherapy + exemestane
perche	ofs + tamoxifen/exemestane vs. ofs + chemotherapy + tamoxifen/exemestane
*Chemotherapy trials*	
MA.21	Phase 3 trial of 1500 node-positive or high-risk node-negative patients Randomized to cef^a^ every 4 weeks × 6 vs. ddEC^b^ + G-csf + epoetin alpha every 2 weeks × 6 vs. AC c every 3 weeks × 4, then T d every 3 weeks × 4
nsabp B-36	Comparing standard ac^c^ × 4 cycles with fec 100 × 6 cycles in node-negative women
nsabp B-38	Goal of 4800 patients with node-positive breast cancer
	tac^e^ every 3 weeks × 6 vs. ddAC^c^ → d every 2 weeks × 4 vs. ddAC c → T d + G f every 2 weeks × 4
*Trastuzumab trials*	
nsabp B-31/NCCTG N9831	Pooled analysis available, but full analysis not available yet, especially concurrent vs. sequential arms in the ncctg trial (expected in 2008)
*Other trials*	
Tailorx Breast Cancer Trial	Will enrol more than 10,000 women with er+/Pr +, her2/*neu*– breast cancer Will examine whether genes that are frequently associated with risk of recurrence can be used to assign patients to the most effective treatment Incorporates a molecular profiling test into clinical decision-making, and thus may spare women unnecessary treatment if chemotherapy is not likely to be of substantial benefit

a Cyclophosphamide 75 mg/m^2^, days 1, 8; epirubicin 60 mg/m^2^, days 1, 8; 5-fluorouracil 500 mg/m^2^.

b Epirubicin 120 mg/m^2^, cyclophosphamide 830 mg/m^2^.

c Doxorubicin 60 mg/m^2^, cyclophosphamide 600 mg/m^2^.

d Paclitaxel 175 mg/m^2^.

e Docetaxel 75 mg/m^2^, doxorubicin 50 mg/m^2^, cyclophosphamide 500 mg/m^2^.

f Gemcitabine 2000 mg/m^2^.

ofs = triptorelin × 5 years, surgical oophorectomy, ovarian radiation; cef = cyclophosphamide, epirubicin, 5-fluorouracil; dd = dose-dense; EC = epirubicin and cyclophosphamide; g-csf = granulocyte colony–stimulating factor; T =tamoxifen; ac = doxorubicin, cyclophosphamide; fec = 5-fluorouracil, epirubicin, cyclophosphamide; tac = docetaxel, doxorubicin, cyclophosphamide; er+ = estrogen-receptor-positive disease; pr+ = progesterone-receptor-positive disease.

**TABLE II:** Characteristics of the adjuvant endocrine trials

*Trial*	*Drug*	*Patients (*n*)*	*Mean age (years)*	*Median follow-up*	*Node+ (%)*	*hr+ (%)*	*Prior chemo (%)*	*Treatment phase*
atac	Anastrozole	9366	64	68	34	84	21	Upfront
big 1–98	Letrozole	8010	61	29	41	100	25	Upfront
MA.17	Letrozole	5187	62	30	46	100	46	Extended 5 years of tamoxifen
IES	Exemestane	4742	64	58	50	81	32	Sequential
arno-95/abcsg-8	Anastrozole	3224	63	30	27	100	0	Sequential
ita	Anastrozole	448	63	36	99.7	88	67	Sequential

Node+ = node-positive disease; hr+ = hormone-receptor-positive disease.

**TABLE III:** Results of adjuvant endocrine trials

		*hr(*p *value)*			
*Trial*	*Treatment*	*dfs*	*os*	*dr*	*Contralateral breast cancer Incidence orhr*
atac	(A) Anastrozole a × 5 years vs. (T) Tamoxifen b × 5 years vs. (A+T) Tamoxifen b + anastrozole a × 5 years	0.83 (0.01)	0.97 (0.7)	0.86 (0.04)	(A) 0.4% (T) 1% (A+T) 0.9%
1–98 big	(T) Tamoxifen b × 5 years vs. (L) Letrozole c × 5 years vs. Tamoxifen b × 2 years → letrozole c × 3 years vs. Letrozole c × 2 years → tamoxifen b × 3 years	0.81 (0.003)	0.86 (0.16)	0.73 (0.001)	(T) 0.7% (L) 0.4%
MA.17	(T) Tamoxifen b × 5 years, then (L) letrozole c × 5 years, or (P) placebo × 5 years	0.57 (0.00008)	0.82 (0.30)	0.60 (0.002)	(P) 1% (L) 0.5%
IES	(T) Tamoxifen b × 5 years vs. (T) Tamoxifen b × 2–3 years, (E) then exemestane d × 2–3 years	0.76 (0.0001)	0.83 (all patients) (0.08) 0.83 (er+/unknown) (0.05)	0.83 (0.03)	hr 0.56 (*p=*0.04)
arno-95/ABCSG-8	(T) Tamoxifen b × 5 years vs. (T) Tamoxifen b × 2 years, then (A) anastrozole a × 3 years	0.60 (0.0018) arno-95 alone 2006 update: 0.53 (0.045) arno-95 alone 2006 update: 0.66 (0.049)	nr (0.16) (T→A) 0.7% arno-95 alone 2006 update: 0.53 (0.045)	nr	(T) 1.1% (T→A) 0.7%
ita	(T) Tamoxifen b × 5 years vs. (T) Tamoxifen b × 2 years, then (A) anastrozole a × 3 years	0.35 (0.001)	7 breast cancer deaths with (T) vs. 4 with (A) (0.1)	0.49 (0.06)	nr

a 1 mg orally, once daily.

b 20 mg orally, once daily.

c 2.5 mg orally, once daily.

d 25 mg orally, once daily.

hr = hormone receptor; dfs = disease-free survival; os = overall survival; dr = distant recurrence; er+ = estrogen-receptor-positive disease; nr = not reported.

**TABLE IV:** Adjuvant chemotherapy studies

*Study*	*Patients (n)*	*Primary endpoint*	*Treatment*	*Results*	*Conclusions*
nsabp-B15	2194 Node+	os	ac^a^ × 4 vs. cmf^b^ × 6 vs. ac^a^ × 4 → 6-month rest → cmf^b^ × 3	62.3% dfs 83% os (3 years)	ac × 4 not superior to cmf × 6
nsabp-B23	2008 Node–/er−	dfs/os	ac^a^ × 4 vs. ac^a^ × 4 + tamoxifen × 5 years vs. cmf^b^ × 6 + tamoxifen × 5 years	dfs: cmf 82.7% vs. ac 82.9% os: cmf 88.5% vs. ac 90.2% (*p*=0.76)	cmf = fec 50
iccg	759 Node+	rfs/os	cmf^b^ × 6 vs. fec 50 c × 6	5-Year os: cmf 77.7% vs. fec 71.5% (*p*=0.96)	
fasg 05	537 >3 LNs+ or 1–3 nodes+ Grades 2/3 er/pr−	dfs/os	fec 50 c × 6 vs. fec 100 d× 6	5-year dfs: fec 50 55% vs. fec 100 66% (*p*=0.03) 5-Year os: fec 50 65% vs. fec 100 77% (*p*=0.007)	fec 100 is better than fec 50
ncic-MA5	710 Node+ pre-/perimenopausal	rfs/os	cmf^b^ × 6 vs. cef^e^ × 6	5-Year rfs: cmf 53% vs. cef 63% (*p*=0.009) 5-Year os: cmf 70% vs. cef 77% (*p*=0.03)	cef better in those with her2/neu overexpression (*p*=0.0307 vs. *p*=0.58 if her2/neu is not overexpressed)
calgb 9344	3121 Node+	dfs/os	ac^a^ × 4 → no treatment vs. → T f × 4 er+ pr+ got 5 years of tamoxifen	5-Year dfs: ac 65% vs. ac-T 70% (*p*=0.0011) 5-Year os: ac 77% vs. ac-T 80% (*p*=0.0098)	Adjuvant paclitaxel improves dfs/os
nsabp -B28	3060 node+	dfs/os	ac^a^ × 4 vs. ac^a^ × 4 → T g × 4	5-Year dfs: ac 72% vs. ac-T 76% (*p*=0.008) 5-Year os: ac 85% vs. ac-T 85% (*p*=0.46)	Addition of Paclitaxel improves dfs but not os
MD Anderson	524	dfs/os	fac^h^ × 8 vs. T i × 4 → fac^i^ × 4	4-Year dfs: fac × 8 83% vs. t-fac 86% (*p*=NS) Similar os	Imbalance in chemotherapy duration may have led to negative results
bcirg 001	1491 Node+	dfs/os	fac^h^ × 6 vs. tac^j^ × 6	At 55 months, dfs: tac 75% vs. fac 68% (*p*=0.001) os: tac 87% vs. fac 81% (*p*=0.008)	tac superior to fac
pacs 01	1999 Node+	dfs/os	fec 100 d × 6 vs. fec 100 d × 3 → D k × 3	5-Year dfs: fec-d 78% vs. fec 73% (*p*=0.01) 5-Year os: fec-d 91% vs. fec 87% (*p*=0.01)	Addition of adjuvant docetaxel in sequence leads to improved dfs and os
US Oncology Trial	1016 Node– (48%) Node+	dfs/os	ac^a^ × 4 vs. tc^l^ × 4	5-Year dfs: tc 86% vs. ac 80% (*p*=0.027) 5-Year os: tc 90% vs. ac 87% (*p*=0.13)	tc results in less nausea/vomiting
CALGB 9741	2005 Node+	dfs/os	A m × 4 → T *n* × 4 → C o × 4 vs. ac^a^ × 4 → T f × 4 (every 14 or 21 days)	4-Year dfs: dose-dense 82% vs. conventional 75% (*p*=0.01) 3-Year os: dose-dense 92% vs. conventional 90% (*p*=0.013)	Dose-dense superior to conventional; concurrent and sequential equal Most of the benefit in ER− population

a Doxorubicin 60 mg/m^2^, cyclophosphamide 600 mg/m^2^, every 3 weeks.

b Cyclophosphamide 100 mg/m^2^, days 1–14; methotrexate 40 mg/m^2^, days 1, 8; 5-fluorouracil 600 mg/m^2^, days 1, 8; every 4 weeks.

c 5-Fluorouracil 500 mg/m^2^, epirubicin 50 mg/m^2^, cyclophosphamide 500 mg/m^2^, every 4 weeks.

d 5-Fluorouracil 500 mg/m^2^, epirubicin (100 mg/m^2^), cyclophosphamide 500 mg/m^2^, every 4 weeks.

e Cyclophosphamide 75 mg/m^2^, days 1–14; epirubicin 60 mg/m^2^, days 1, 8; 5-fluorouracil 500 mg/m^2^, days 1, 8; every 4 weeks.

f Paclitaxel 175 mg/m^2^ over 3 hours, every 3 weeks.

g Paclitaxel 225 mg/m^2^ over 3 hours, every 3 weeks.

h 5-Fluorouracil 500 mg/m^2^, doxorubicin 50 mg/m^2^, cyclophosphamide 500 mg/m^2^, every 3 weeks.

i Paclitaxel 250 mg/m^2^ over 24 hours, every 3 weeks.

j Docetaxel 75 mg/m^2^, doxorubicin 50 mg/m^2^, cyclophosphamide 500 mg/m^2^, every 3 weeks.

k Docetaxel 100 mg/m^2^, every 3 weeks.

l Docetaxel 75 mg/m^2^, cyclophosphamide 600 mg/m^2^, every 3 weeks.

m Doxorubicin 60 mg/m^2^, every 3 weeks.

n Paclitaxel 175 mg/m^2^, every 3 weeks.

o Cyclophosphamide 600 mg/m^2^, every 3 weeks.

os = overall survival; ac = doxorubicin, cyclophosphamide; dfs = disease-free survival; cmf = cyclophosphamide, methotrexate, 5-fluorouracil; fec = 5-fluorouracil, epirubicin, cyclophosphamide; Node– = node-negative disease; er− = estrogen-receptor-negative disease; rfs = recurrence-free survival; Node+ = node-positive disease; LN = lymph node; pr− = progesterone-negative disease; cef = cyclophosphamide, epirubicin, 5-fluorouracil; T = tamoxifen; pr+ = progesterone-positive disease; fac = 5-fluorouracil, doxirubicin, cyclophosphamide; tac = docetaxel, doxorubicin, cyclophosphamide; D = docetaxel; A = doxorubicin; C = cyclophosphamide.

**TABLE V:** Adjuvant trastuzumab trials

					*hr*	
*Trial*	*Eligibility*	*Patients (n)*	*Regimen*	*Median follow-up*	*dfs*	*os*
nsabp B-31/ncctg N-9831 (combined analysis concurrent vs. no trastuzumab)	her2+ Node+/high-risk node– (Node+/high-risk node– (ncctg only)	1736/1615 Total=3351	nsabp B-31: ac^a^ × 4 → T b × 4 alone or T b × 4 and trastuzumab c (H) × 1 year ncctg N-9831: ac^a^ × 4 → T d × 12 weeks or ac^a^ × 4 → T d × 12 weeks → H c × 52 weeks or ac^a^ × 4 → T d + H c × 12 weeks → H c × 40 weeks	2 Years	0.50 (*p*=0.0005)	0.67 (*p*=0.015)
hera	her2+, node+/high-risk node–, no lvef post-chemo	5090	Any Chemotherapy then Observation or H e × 1 year or H e × 2 years	2 Years	0.63 (*p*<0.0001)	0.63 (*p*=0.0051)
bcirg 006	her2+, node+/high-risk node–, no lvef	3222	ac^a^ × 4 → D f × 4 or ac^a^ × 4 → D f + H c × 4 → H e × 40 weeks or D^g^ C^h^ H^e^ × 6	23 Months	0.49 (ac→dh) (*p*<0.0001) 0.61 (dch) (*p*=0.0002)	nr
FinHer	her2+, node+/high-risk node–	1010	D f every 3 weeks × 3 → fec^i^ 60 × 3 or V j every week × 8 → fec^i^ 60 × 3 then If her2+, randomized to no further treatment or H c every week × 9 weeks	36 Months	0.42 (*p*=0.01)	0.41 (*p*=0.07)

a Doxorubicin 60 mg/m^2^, cyclophosphamide 600 mg/m^2^, every 3 weeks.

b Paclitaxel 175 mg/m^2^ over 3 hours, every 3 weeks.

c Trastuzumab 4 mg/m^2^ load, then 2 mg/m^2^, every week.

d Paclitaxel 80 mg/m^2^, every week.

e Trastuzumab 8 mg/m^2^ load, then 6 mg/m^2^, every 3 weeks.

f Docetaxel 100 mg/m^2^, every 3 weeks.

g Docetaxel 75 mg/m^2^, every 3 weeks.

h Carboplatin auc 6, every 3 weeks.

i 5-Fluorouracil 600 mg/m^2^, epirubicin 60 mg/m^2^, cyclophosphamide 600 mg/m^2^.

j Vinorelbine 25 mg/m^2^, every week.

hr = hormone receptor; dfs = disease-free survival; os = overall survival; ac = doxorubicin, cyclophosphamide; T = tamoxifen; node+ = node-positive disease; node– = node-negative disease; H = trastuzumab (Herceptin); lvef = left ventricular ejection fraction; D = docetaxel; dh = docetaxel, Herceptin; dch = docetaxel, carboplatin, Herceptin; C = Carboplatin; fec = 5-fluorouracil, epirubicin, cyclophosphamide; V = vinorelbine.
